# Expression of apoptosis-related markers and clinical outcome in patients with advanced colorectal cancer

**DOI:** 10.1054/bjoc.2000.1658

**Published:** 2001-03

**Authors:** A Paradiso, G Simone, M De Lena, B Leone, C Vallejo, J Lacava, S Dellapasqua, M G Daidone, A Costa

**Affiliations:** Clinical Experimental Oncology Laboratory; Histopathology Service; Medical Oncology Division, Oncology Institute, Bari, Italy; Grupo Oncologico Cooperative del Sur, Neuquen, Argentina; Determinants of Prognosis and Treatment Response Unit, Department of Experimental Oncology, Istituto Nazionale per lo Studio e la Cura dei Tumori, Milan, Italy

**Keywords:** bax expression, bcl-2 expression, advanced colorectal cancer, response to treatment, p53 expression, apoptotic index

## Abstract

The clinical relevance of bax and bcl-2 protein expression has been investigated in 84 patients with recurrent or metastatic colorectal cancer submitted to a chemotherapy regimen including methotrexate and fluorouracil/leucovorin. Cytoplasmic immunostaining of bax and bcl-2 was present in 65.5% and 38%, respectively, of the tumours. No association was found between bax and bcl-2 or between p53 and bax or bcl-2 protein expression. Moreover, the biomarkers were unrelated to patient and tumour characteristics known to affect the clinical outcome of colorectal cancer patients. In general, the apoptosis-related markers did not appear indicative of short- and long-term clinical response nor of prognosis. Bcl-2-negative lesions were more frequent among patients who reached an objective clinical response, which is in agreement with previously reported data regarding other tumour types. When the interrelationship between p53 and bax expression was examined, a better response rate (40%) was found for patients whose tumours did not express p53 and bax, and a better prognosis (2-year probability of overall survival 75%) for patients with p53-positive and bax-negative tumours. In the present series of patients with advanced colorectal cancer submitted to systemic chemotherapy we did not find a clear association between expression of apoptosis-related markers and clinical outcome, even in the subset of patients in which the apoptotic index as determined by the TUNEL approach was investigated. © 2001 Cancer Research Campaign http://www.bjcancer.com


*British Journal of Cancer* (2001) **84**(5), 651–658
© 2001 Cancer Research Campaign
doi: 10.1054/ bjoc.2000.1658, available online at http://www.idealibrary.com on IDEAL

http://www.bjcancer.com

### Expression of apoptosis-related markers and clinical outcome in patients with advanced colorectal cancer

**A Paradiso[1], G Simone[2], M De Lena[3], B Leone[4], C Vallejo[4], J Lacava[4], S Dellapasqua[5], MG Daidone[5] and A Costa[5]**

[1]Clinical Experimental Oncology Laboratory, [2]Histopathology Service, [3]Medical Oncology Division, Oncology Institute, Bari, Italy; [4]Grupo Oncologico Cooperative del Sur, Neuquen, Argentina; [5]Determinants of Prognosis and Treatment Response Unit, Department of Experimental Oncology, Istituto Nazionale per lo Studio e la Cura dei Tumori, Milan, Italy

**Summary** The clinical relevance of bax and bcl-2 protein expression has been investigated in 84 patients with recurrent or metastatic colorectal cancer submitted to a chemotherapy regimen including methotrexate and fluorouracil/leucovorin. Cytoplasmic immunostaining of bax and bcl-2 was present in 65.5% and 38%, respectively, of the tumours. No association was found between bax and bcl-2 or between p53 and bax or bcl-2 protein expression. Moreover, the biomarkers were unrelated to patient and tumour characteristics known to affect the clinical outcome of colorectal cancer patients. In general, the apoptosis-related markers did not appear indicative of short- and long-term clinical response nor of prognosis. Bcl-2-negative lesions were more frequent among patients who reached an objective clinical response, which is in agreement with previously reported data regarding other tumour types. When the interrelationship between p53 and bax expression was examined, a better response rate (40%) was found for patients whose tumours did not express p53 and bax, and a better prognosis (2-year probability of overall survival 75%) for patients with p53-positive and bax-negative tumours. In the present series of patients with advanced colorectal cancer submitted to systemic chemotherapy we did not find a clear association between expression of apoptosis-related markers and clinical outcome, even in the subset of patients in which the apoptotic index as determined by the TUNEL approach was investigated.
© 2001 Cancer Research Campaign http://www.bjcancer.com

**Keywords**: bax expression; bcl-2 expression; advanced colorectal cancer; response to treatment; p53 expression; apoptotic index

Apoptosis is a form of cell death which is regulated at the gene level and plays a central role in cell number control during embryonic development and organogenesis; it determines the cellular response to various physiological situations in adult tissues and during pathophysiological conditions including neoplastic transformation. In addition, defects in the apoptotic pathway may represent a critical element in the progression of neoplastic disease and may also concur to determine treatment efficacy at the cellular level. In fact, many of the effects of the chemical and physical agents that are commonly used in the treatment of human malignancies are mediated by induction of apoptosis (Eastman, 1990; Dive and Hickman, 1991; Schmitt and Lowe, 1999) and thus rely at least in part on the same biochemical mechanisms involved in physiological cell death control. As a consequence, genetic alterations that prevent or delay normal cell turnover may also be responsible for treatment inefficacy in tumour cells.

Bax and bcl-2 are members of the large family of proteins that constitute a critical intracellular checkpoint within a common cell death pathway involved in determining the susceptibility of cells to undergo apoptosis. This checkpoint is controlled by the ratio between promoters (i.e. bax) and inhibitors (i.e. bcl-2) of cell death and although these molecules compete through dimerization, extensive mutational studies have not been able to establish whether their function is interdependent or whether either agonists or antagonists are dominant (Sionov and Haupt, 1999).

Bcl-2 protein expression or gene activation have been associated with poor response to therapy and/or shorter disease-free survival in some groups of patients with lymphomas (Yunis et al, 1989), leukaemias (Campos et al, 1993), prostate cancer (McDonnel et al, 1992) and breast cancer (Bonetti et al, 1998; Daidone et al, 1999). In head and neck tumours on the other hand bcl-2 positivity proved to be either highly indicative (Gasparini et al, 1995, Homma et al, 1999) or independent (Costa et al, 1998) of the response to treatment. A poor response to chemotherapy and short survival was observed in a subgroup of patients with metastatic breast cancer whose tumours showed reduced bax immunostaining (Krajewski et al, 1995). Similarly, it was shown that high bax expression in ovarian cancer was associated with a significant increase in the percentage of complete remissions after first-line chemotherapy including paclitaxel and a platinum analogue, and also with an improvement in survival (Tai et al, 1998), although such findings are not universal (Silvestrini et al, 1998).

In patients with advanced or metastatic colorectal cancer chemotherapy with fluorouracil plus leucovorin (5-FU/LV) provides an overall response rate of approximately 25% with a limited effect on survival (Clark, 1997). As a consequence, alternative agents and new treatment strategies or combinations are being investigated. Research efforts have increased our understanding of how anticancer agents mediate their effects, and

*Received 8 May 2000*
*Revised 14 November 2000*
*Accepted 30 November 2000*

*Correspondence to:* A Paradiso, Clinical Experimental Oncology Laboratory, National Oncology Institute, Via Amendola, 209, 70126 Bari, Italy.
E-mail: anpara@tin.it specific targets to inhibit the growth or dissemination of this tumour type have been identified. However, in advanced colorectal cancer the search for indicators of response to chemotherapy still represents a valuable effort to potentially improve the therapeutic decision-making following failure to first-line therapies.

We have analysed the expression of bax and bcl-2 in a group of 84 patients with recurrent or metastatic colorectal cancer, homogeneously treated with the same polychemotherapy regimen, in an attempt to elicit the role of apoptosis-related markers as indicators of clinical outcome. Moreover, the expression pattern of bax and bcl-2 was analysed in comparison with that of p53, which had previously been determined in the present case series (Paradiso et al, 1996), and their interactions were investigated in relation to clinical outcome. Since morphological analysis appears the unequivocal gold standard (Hall, 1999) for the assessment and quantitation of apoptosis, in a subset of 48 patients of the present series we determined the apoptotic index (AI) as defined by the TUNEL technique (Gavrieli et al, 1992). The relationship between AI and the expression of the apoptosis-related proteins bax, bcl-2 and p53 was examined and its potential clinical role in terms of response to treatment, time to progression and overall survival was analysed.

### MATERIAL AND METHODS

### Patients and treatment

Treatment modalities and inclusion criteria of the patients involved in this study have been described in detail elsewhere (Machiavelli et al, 1991; Paradiso et al, 1996). All patients had advanced disease at first diagnosis or recurrent disease and had not been previously treated with any systemic chemotherapy. Briefly, formalin-fixed, paraffin-embedded samples of surgically resected primary colorectal cancer from 84 patients participating in the treatment protocols of the institutions of GOCS-Argentina (Machiavelli et al, 1991) were available for retrospective biological characterization. Patients received methotrexate (MTX) at 200 mg/m$^2$ intravenously (i.v.) by push injection followed by 5-FU at 1200 mg/m$^2$ as a continuous i.v. infusion over 2 h; 24 h after MTX administration all patients received 25 mg LV every 6 h for a total of 8 doses. The treatment schedule was repeated every 15 days until disease progression, severe toxicity or death. Patients were considered evaluable for response when they had received at least 2 chemotherapy cycles. Standard UICC criteria were used for evaluation of clinical response, and objective regression (OR) included complete remission (CR) and partial response (PR).

### Immunohistochemical staining for bax and bcl-2 proteins

Immunohistochemical staining was performed on tissue sections of formalin-fixed, paraffin-embedded surgical specimens from primary tumours. The 4-μm-thick slices were deparaffinized in xylene, dehydrated with graded ethanol and submitted to microwave irradiation for 10 min in 10 mM citrate buffer (pH 6.0) to detect masked and unmasked antigen sites.

#### *bax expression*

The microwave-treated sections were incubated for 2 h at 4˚C with a 1:400 dilution of polyclonal rabbit antibody bax N-20 (Santa Cruz Biotechnology Inc, Santa Cruz, CA) as previously described (Costa et al, 1998). After incubation the specimens were treated with a biotinylated anti-rabbit immunoglobulin and processed using immunoperoxidase staining (Vectastain ABC Kit). The quality of the bax antiserum used batch was assessed by Western blot analysis on human colorectal cancer cell lines.

#### *bcl-2 expression*

The microwave-treated sections were incubated for 1 h at room temperature in a humidified atmosphere with a 1:40 dilution of monoclonal mouse anti-human bcl-2 oncoprotein (clone 124; Dakopatts, Copenhagen, Denmark), as previously described (Costa et al, 1998). As with bax, the ABC immunoperoxidase system was used.

Colorectal cancers with high bax or bcl-2 immunoreactivity were used as positive controls, whereas negative controls for the markers were obtained by omission of the primary antibody. Positivity for bax and bcl-2 was present when unequivocal brown staining was observed in tumour cells at the cytoplasmic level, and semiquantitative measurement was performed by scoring a total of 1000 to 3000 tumour cells of the same sample.

#### *p53 expression*

In the present series p53-protein expression had been determined as part of a previous study, which includes a detailed description of the method (Paradiso et al, 1996). Briefly, microwave-treated sections were incubated for 1 h at room temperature with a 1:50 dilution of PAb1801 monoclonal antibody (Oncogene Science, Inc, Manhasset, NY). The monoclonal antibody, raised against human p53, recognizes wild-type and mutant forms of the p53 protein.

Evaluation of the biomarkers was performed independently by two observers who were unaware of the clinical outcome. The determination of bcl-2 and p53 expression was performed within National Quality Control Programs (Finalized Project 98/90, Ministry of Health, Italy). In the overall series of 84 cases, information was available on bax and bcl-2 protein expression for all tumours, whereas p53 protein expression determination was available for 83 cases.

### Apoptotic index assessment

Apoptotic cells and apoptotic bodies were detected in a subset of 48 cases with adequate tumour material following previous determinations from the overall series of 84 tumours using the *In Situ* Cell Death Detection Kit, POD (Boehringer Mannheim GmbH, Biochemica, Germany), an indirect TUNEL labelling assay. Briefly, deparaffinized and rehydrated sections were digested with protease K (1 μg ml$^{-1}$ in PBS; Sigma-Aldrich S.r.l., Milan, Italy) for 15 min at 37˚C and washed. After quenching in 3% hydrogen peroxide for 10 min, washing with PBS, and adding the equilibration buffer for 10 min, the sections were incubated at 37˚C for 1 h with terminal deoxynucleotidyl transferase enzyme. After the reaction had been stopped by placing the sections in stop/wash buffer and washing them, anti-digoxigenin-peroxidase was added to the slides. Finally, the slides were washed with PBS, stained with diaminobenzidine (Dakopatts) substrate, and counterstained with haematoxylin. A positive control was prepared by nicking DNA with DNase I (1 μg ml$^{-1}$; Sigma-Aldrich S.r.l.) for the first staining procedure. A specimen known to be positive for apoptotic cells was used as positive control for subsequent

*© 2001 Cancer Research Campaign*

staining. Substitution of terminal deoxynucleotidyl transferase with distilled water was used as negative control. AI was expressed as the ratio of positive-staining tumour cells and bodies to all tumour cells and was given as a percentage for each case. Necrotic areas and positive cells located in the stroma and lumen were avoided as they could have originated from other cell types. A minimum of 3000 cells was counted using 400-fold magnification. Positive-staining tumour cells with the morphological characteristics of apoptosis were identified using standard criteria including chromatin condensation, nuclear disintegration, and formation of crescentic caps of condensed chromatin at the nuclear periphery.

### Statistical analysis

For basic analysis, bax, bcl-2 and p53 expression and AI were considered as continuous variables and their relationship was investigated by Spearman's regression coefficient (rs). The association between bax and bcl-2 expression, AI and clinicopathological features was assessed by means of Wilcoxon's rank-sum test.

When bax, bcl-2 and p53 expression was related to clinical outcome, biomarkers were considered as dichotomous variables by taking as the cutoff for negativity/positivity the value of 5% stained cells. For p53 in colorectal cancer such a cutoff value provided the best concordance between mutational status and protein accumulation evaluated by immunohistochemistry (Costa et al, 1995). The median value of 0.6% was used as a cutoff between low and high AI. The association between biomarkers, AI and clinical response was evaluated by the chi-square test, continuity adjusted when appropriate.

Time to progression (TTP) and overall survival (OS) were calculated as the interval between the date of initiation of treatment and the date of first progression (local progression or distant metastasis) or the date of death (or of the last follow-up for censored observations), and were estimated by the Kaplan–Meier product-limit method. The influence of the biomarkers on TTP and OS was evaluated by fitting a Cox regression model. Hazard ratios (HR) and their 95% confidence limits (CL) were determined using the putative best prognostic category as a reference. At the time of the present analyses, 67 patients progressed or developed distant metastases and 53 patients died. The median OS for surviving patients was 13 months (range, 1 to 52 months).

### RESULTS

### Immunostaining of apoptosis-related markers

Positive immunoreactivity for bax and bcl-2 was localized in the cytoplasm of tumour cells, and most tumours that were scored as positive showed homogenous intensity of immunostaining. Bax or bcl-2 immunostaining was present in residual normal epithelial cells or infiltrating lymphocytes and these non-malignant cells were an internal positive control to verify adequate specimen preservation in non-immunoreactive malignant tissues. 55 of the 84 tumours (65.5%) showed more than 5% bax-immunopositive cells ($bax^+$), and the median fraction of bax-expressing cells in $bax^+$ tumours was 79% (range, 10–100%). For bcl-2 protein expression, 38% of tumours (32/84 cases) showed more than 5% bcl-2-positive cells ($bcl\text{-}2^+$), and the median fraction of bcl-2 expressing cells in $bcl\text{-}2^+$ tumours was 40% (range, 10–80%). In the 83 cases for which also p53 protein expression had been determined, nuclear immunoreactivity in more than 5% of tumour cells ($p53^+$) was observed in 39% of tumours (32/83 cases), with a median value of 40% immunoreactive cells in $p53^+$ cases (range, 10–100%). Apoptotic cells and apoptotic bodies were detected in all 48 colorectal caracinomas examined by in situ labelling, and the median AI was 0.6% (range, 0.02–5.9%).

Bax, bcl-2 and p53 proved to be expressed independently of each other, as shown by the very low correlation coefficients (bax *vs* bcl-2, $r_s = -0.02$; bax *vs* p53, $r_s = 0.13$; bcl-2 *vs* p53, $r_s = 0.18$). In addition, a similar distribution of bax and bcl-2 immunoreactive cells was observed as a function of p53 expression. In fact, the same median frequency of $bax^+$ cells (65%) was observed in $p53^-$ and $p53^+$ tumours, and bcl-2 immunoreactivity was not statistically different in the $p53^-$ (median $bcl\text{-}2^+$ cells, 30%) and $p53^+$ (median $bcl\text{-}2^+$ cells, 60%) tumour subsets. Moreover, AI was unrelated to bax, bcl-2 and p53 expression.

In the present series of patients, as previously reported for p53 protein (Paradiso et al, 1996), the expression of the two apoptosis-related proteins was independent of those patient and tumour features that are conventionally indicative of clinical outcome for this neoplasm, i.e. patient age, site of the primary tumour, time of disease (first diagnosis *vs* recurrence), type of metastases (site and number) and histological grade. Also, AI was not significantly associated with any of the clinicopathological factors.

### Clinical outcome as a function of biomarkers

80 of the 84 patients who entered the study received more than 2 cycles of chemotherapy; an OR was reached in 25% of these cases whereas 35% (28/80) had progressive disease, and the remaining 32 patients had stable disease during treatment. At 24 months from the start of treatment the probability of being free of progression was only 2% and the median time to progression was 11, 8 and 3 months, respectively, for patients having OR, stable disease or disease progression during treatment. The probability of being alive at 24 months from the start of treatment was 39%, 38% and 0%, respectively, for the 3 subsets of patients.

In Table 1 the response rates are analysed as a function of the different bax and bcl-2 immunocytochemical patterns. Objective clinical response was not related to the expression of bax or bcl-2, even though 15 of the 20 patients who achieved an OR had a bcl-2-tumour. When the combined bax/bcl-2 patterns of immunostaining were examined, the two markers again did not appear associated with the response rate following MTX and 5-FU/LV treatment (data not shown). In the subset of patients for whom AI was determined, a (not statistically significant) trend towards an association between apoptosis and response to treatment was observed (Table 1). A high AI ($\geq 0.6\%$) was observed in 7 of the 10 patients achieving objective response, whereas a low AI ($< 0.6\%$) was observed in 10 of 15 patients with progressive disease.

Univariate analysis showed that 2-year TTP and OS were independent of apoptosis-related markers (Figure 1) and the survival curves were almost superimposable for subsets defined according to the expression of bax or bcl-2, except for patients with $bax^+$ tumours, whose median survival time was slightly shorter than that observed for patients with bax- tumours (9 *vs* 14 months). The trend in favour of an association between AI and response to treatment was not reflected by the 2-year clinical outcome. In fact, the presence of high or low AI did not segregate subsets of patients at different prognosis in terms of TTP and OS (data not shown).

*© 2001 Cancer Research Campaign* 

**Table 1** Relationship between response to polychemotherapy including MTX and 5-FU/LV and apoptosis-related markers in patients with advanced colorectal adenocarcinomas

| | Total | Objective regression No. of cases (%) | Stable disease No. of cases (%) | Progressive disease No. of cases (%) |
|---|---|---|---|---|
| bax expression[a] | | | | |
| Negative | 28 | 9 (32%) | 11 (39%) | 8 (29%) |
| Positive | 52 | 11 (21%) | 21 (40%) | 20 (39%) |
| bcl-2 expression[b] | | | | |
| Negative | 51 | 15 (30%) | 20 (39%) | 16 (31%) |
| Positive | 29 | 5 (18%) | 12 (41%) | 12 (41%) |
| AI[c] | | | | |
| <0.6% | 23 | 3 (14%) | 10 (43%) | 10 (43%) |
| ≥0.6% | 23 | 7 (30%) | 11 (48%) | 5 (22%) |

[a]chi square = 1.393, $P = 0.50$; [b]chi square = 1.646, $P = 0.44$; [c]chi square = 3.314, $P = 0.19$

**Table 2** Clinical outcome as a function of bax and bcl-2 in advanced colorectal adenocarcinomas with different p53 expression

| | % of OR (no. cases) | | 2-yr probability of OS | |
|---|---|---|---|---|
| | p53– | p53+ | p53– | p53+ |
| bax– | 40 (8/20) | 13 (1/8) | 21 | 75 |
| bax+ | 21 (6/29) | 22 (5/23) | 33 | 25 |
| bcl-2– | 34 (11/32) | 21 (4/19) | 29 | 27 |
| bcl-2+ | 18 (3/17) | 17 (2/12) | 28 | 30 |

OR, objective regression; OS, overall survival

Furthermore, the analysis of the potential role of apoptosis-related markers on clinical outcome was performed by considering in association p53 and bax or bcl-2 expression. Since these results were derived from subset analyses, comparisons between the proposed subgroups were meant to be descriptive, preliminary and hypothesis-generating only. The highest response rates (Table 2) were observed within the subgroup of patients with p53- tumours which did not express bax or bcl-2 proteins (40% and 34%, respectively). With respect to OS, p53 protein expression was not indicative of clinical outcome for this selected series of patients with advanced colorectal cancer (Paradiso et al, 1996), although a combined analysis that considered bax and bcl-2 expression identified a small subgroup of 8 patients with $bax^-/p53^+$ tumours with a 75% probability of being alive at 2 years as compared to the lower probabilities found for the remaining subgroups of patients (Table 2).

### DISCUSSION

In the present study we evaluated the association between clinical outcome and the expression of markers related to the apoptotic process in a group of patients with recurrent or metastatic colorectal adenocarcinoma previously characterized for p53 and treated with a polychemotherapy regimen that included MTX and 5-FU/LV. Among the different approaches to evaluate cell susceptibility to the activation of apoptosis, we focused on the expression of two negative and positive regulatory proteins, bcl-2 and bax, whose immunocytochemical determination proved to be feasible on archival specimens and associated with tumour progression and treatment response in different clinical situations involving various tumour types (Krajewski et al, 1995; Barretton et al, 1996; Apolinario et al, 1997; Bonetti et al, 1998; Silvestrini et al, 1998; Tai et al, 1998; Daidone et al, 1999; Ogura et al, 1999; Sturm et al, 1999). Bax represents a pro-apoptotic member of the bcl-2 family which controls important checkpoints during the apoptotic process and whose transcriptional activation is induced by wild-type p53. Thus, cancer may arise through selective loss of an apoptotic pathway as a result of the replication of cells that survived DNA damage or other severe intracellular injury. This could explain the reason why cancer cells arising in this way are resistant to many cytotoxic agents because of loss of critical functions in the control of cell number.

To our knowledge only two studies have evaluated the prognostic relevance of the apoptosis promoter bax in patients with resectable primary colorectal cancer (Ogura et al, 1999) and liver metastases from colorectal cancer (Sturm et al, 1999) submitted to surgery without any adjuvant treatment. In both studies bax expression and/or mutational status proved to be significantly related to clinical outcome. In fact, in these studies involving 58 (Ogura et al, 1999) and 41 (Sturm et al, 1999) patients, those with high bax protein expression had a longer survival than patients with low bax expression, and this finding was especially evident for patients with wild-type p53 liver metastases (Sturm et al, 1999). However, in a group of patients submitted to preoperative radiochemotherapy for locally advanced rectal carcinoma Tannapfel et al (1998) did not find any correlation between the level of bax expression and the degree of clinical-to-pathological downstaging or the frequency of tumour recurrence. In the present study, for the first time in patients with metastatic colorectal cancer submitted to a polychemotherapy regimen, the predictive role of the pro-apoptotic protein bax and the anti-apoptotic protein bcl-2 was analysed in terms of response to treatment and overall survival. In our series of metastatic and recurrent cancer patients, bax protein expression itself did not appear to divide patients into different groups with respect to clinical disease course. Different explanations might account for this finding: 1) treatment might mask the clinical role of bax expression; 2) the aggressive behaviour of advanced disease prevails over the relevance of biological markers for tumour progression or treatment response; 3) the multifactorial nature of intrinsic resistance to antimetabolites and the complex cascade of intracellular signals controlling the activation and progression of the apoptotic programme make it inappropriate to ascribe a great deal of functional significance on the basis of the evaluation of a single gene product such as bax. The last finding prompted us to investigate the information provided by bax, an apoptosis promoter, with that provided by bcl-2, an apoptosis suppressor.

*© 2001 Cancer Research Campaign*

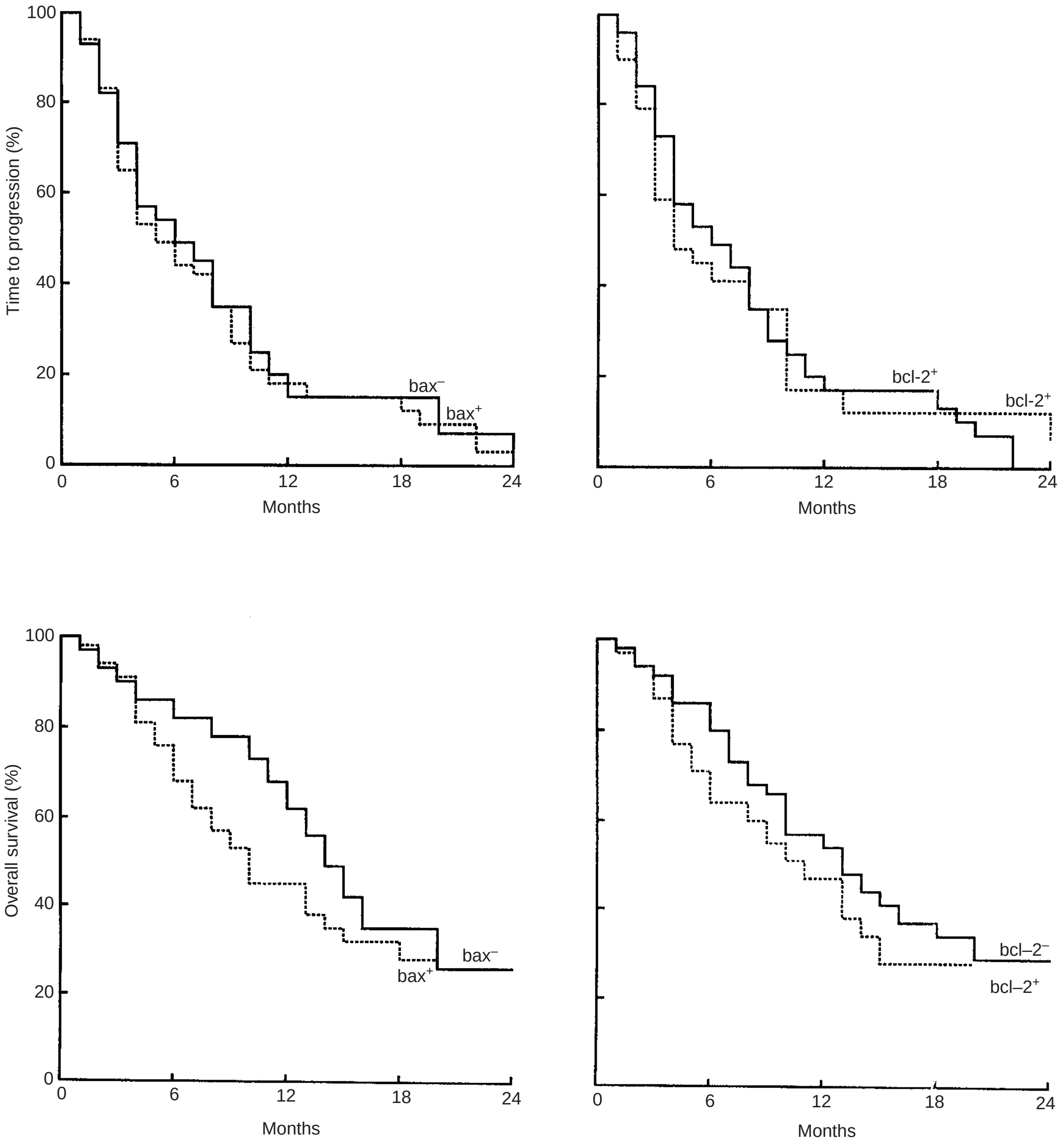

**Figure 1** Clinical outcome, i.e., time to progression (TTP) and overall survival (OS), in patients with advanced colorectal cancer as a function of bax (log-rank test: TTP, $P = 0.79$; OS, $P = 0.30$) and bcl-2 (log-rank test: TTP = 0.96; OS, $P = 0.40$) protein expression

Previous reports on colorectal carcinoma established an association between bcl-2 positive staining and good prognosis (Ofner et al, 1995; Baretton et al, 1996), suggesting that bcl-2 promoted cell survival in slowly growing tumours, which was recently confirmed by Ogura et al (1999) and is in keeping with results obtained in other solid tumours (Silvestrini et al, 1994). All these studies, however, referred to patients with operable colorectal cancer submitted to surgery as the only therapeutic approach. By contrast, in the present series of advanced colorectal patients bcl-2 expression was not indicative of clinical outcome, which is in agreement with the results of a previous study on liver metastases from colorectal cancer (Costa et al, 1997). The only clinically relevant observation was the high objective regression rate observed in patients with bcl-2-negative lesions, in agreement with data reported in other solid tumours such as prostate cancer (McDonnell et al, 1992) and breast cancer (Bonetti et al, 1998), and in systemic diseases such as lymphomas (Yunis et al, 1989) and leukaemias (Campos et al, 1993).

In in vitro experiments high endogenous levels and gene transfer-mediated overexpression of bax have been correlated with increased sensitivity to apoptosis induced by chemotherapeutic drugs (Bargou et al, 1995, 1996). Conversely, gene transfer-mediated elevations in bcl-2 have been shown to promote in vitro resistance to a number of drugs, including anthracyclines (Teixeira et al, 1995) and some antimetabolites (Orlandi et al, 1999). Thus, if one considers the combined pattern of bax/bcl-2 expression one would expect tumours with high bax and low bcl-2 expression to exhibit a higher response rate or a more favourable outcome

*© 2001 Cancer Research Campaign* 

following chemotherapy, as was also observed in other tumour types (Krajewski et al, 1995; Daidone et al, 1999). However, this hypothesis was not supported by the results obtained in the present series of patients, which would suggest that in intrinsic chemoresistance of colorectal cancer the contribution of drug-induced damage to the activation of apoptosis needs to be investigated at different levels in pre- and post-treatment specimens, possibly extending the research to the detection of proteins that can influence apoptosis in certain instances through positive or negative regulation.

In our study, contrary to what was observed in other tumour types (Silvestrini et al, 1994; Krajewski et al, 1995; Apolinario et al, 1997), no relationship was found between p53 and bax or bcl-2 protein expression, i.e., cases not expressing p53 did not present any clear upregulation of bax or downregulation of bcl-2. This finding could imply that bax or bcl-2 might be regulated by mechanisms other than p53, as already reported with regard to colorectal tumours (De Angelis et al, 1998) and other tumour types including breast cancer (Daidone et al, 1999), non-small-cell lung cancer (Apolinario et al, 1997), squamous cell carcinoma of the larynx (Fracchiolla et al, 1999) and of the oral cavity (Costa et al, 1998). Generally, no relationship was observed between clinical outcome and p53/bax or p53/bcl-2 staining patterns, except for a high response rate (42%) in patients with $p53^-$/$bax^-$ tumours. This as well as other findings derived from subset analysis should be considered as hypothesis generating and therefore need to be validated. However, the paradoxical observation of loss of bax correlated with a trend towards a better prognosis could be the consequence of the potential presence of a microsatellite mutator phenotype (MMP). In fact, bax expression can be lost upon frameshift mutations in a tract of 8 deoxyguanosines in the bax gene, frequently present in MMP (Rampino et al, 1997; Abdel-Rahman et al, 1999). It has been reported that the MMP in colorectal cancer is associated with response to 5-FU treatment (Nelson, 1998), and that patients with mismatch repair deficiency have a better clinical outcome (Bubb et al, 1996; Forster et al, 1998; Lukish et al, 1998; Liang et al, 1999).

Since intrinsic drug resistance may be the biological equivalent of resistance to apoptosis induction, in addition to the biomarkers studied we examined the apoptotic index with the TUNEL method in order to detect apoptotic cells in a subset of the present series of tumours. No significant association was found between AI and the expression of the proteins studied, as was also previously reported in colorectal carcinoma by other authors (Takano et al, 1996; Matsuura et al, 2000; Tenjo et al, 2000), our results could not confirm the significance of the p53 gene status in the induction of apoptosis in colorectal carcinomas. This might have been partly due to the small number of cases examined, or it might imply the predominance of a p53-independent pathway in the induction of apoptosis in human colorectal carcinomas. Again, AI did not exhibit the expected correlation with bax and bcl-2 protein expression, as was also reported for gastric (Sugamura et al, 1997) and colorectal (Langlois et al, 1997) cancers. However, these findings are in keeping with the hypothesis that in a proportion of colorectal cancer cases the bcl-2 proto-oncogene expression may be downregulated at a post-transcriptional level (Berney et al, 2000) since the expression of bcl-2 protein was gradually and significantly lost during the progression from moderately dysplastic adenoma to primary colorectal carcinoma; conversely, the cellular expression of bcl-2 mRNA gradually increased during the successive steps of carcinogenesis.

A prognostic role of the AI has been postulated for patients with colorectal cancer, underlying an aggressive pattern in presence of a low frequency of apoptotic cells (Langlois et al, 1997; Kawasaki et al, 1998; Sugamura et al, 1998; Tenjo et al, 2000). It was recently demonstrated that preoperative treatment with tegafur (a prodrug of 5-FU) (Matsuura et al, 2000) or 5-FU (Yamane et al, 1999) enhances apoptosis in human colorectal carcinoma, and that an increase in the apoptotic index after short-term cytostatic treatment is associated with objective responses in patients with rectal cancer (Farczadi et al, 1999). Similarly, we observed a direct relation between AI and response to a polychemotherapy regimen including 5-FU, suggesting that the morphological evaluation of the apoptotic status may provide information on treatment response. This finding is lost on the long-term clinical outcome, but it is worth mentioning that in advanced and metastatic disease the intrinsic aggressive phenotype might overcome the predictive/prognostic role of single biomarkers.

In conclusion, in the present series of patients with metastatic colorectal cancer we did not find clinically relevant associations between markers favouring, delaying or controlling apoptosis such as bax, bcl-2 and p53, and response to a polychemotherapy regimen including MTX and 5-FU/LV. It must be pointed out, however, that in spite of objective clinical responses achieved in 25% of cases, almost all patients developed disease progression within 2 years from the start of treatment, which emphasizes the known aggressiveness and resistance to chemotherapy of this disease at the advanced stages. To overcome the present limitations of commonly used drug-resistance markers which do not satisfactorily predict the clinical outcome of specific subsets of patients, much effort is needed to better identify specific markers of drug action. In this respect promising results seem achievable by analysis of the expression of thymidylate synthase, a crucial enzyme for de novo synthesis of thymidine which is specifically inhibited by 5-FU (Johnston et al, 1991; Paradiso et al, 2000).

### REFERENCES

Abdel-Rahman WM, Georgiades IB, Curtis LJ, Arends MJ and Wyllie AH (1999) Role of BAX mutations in mismatch repair-deficient colorectal carcinogenesis. *Oncogene* **18**: 2139–2142

Apolinario RM, Van Der Valk P, De Jong JS, Deville W, Van Ark-Otte J, Dingemans AMC, Van Mourik JC, Postmus PE, Pinedo HM and Giaccone G (1997) Prognostic value of the expression of p53, bcl-2 and bax oncoproteins, and neovascularization in patients with radically resected non-small-cell lung cancer. *J Clin Oncol* **15**: 2456–2466

Barretton BG, Diebold J, Christoforis G, Vogt M, Müller C, Dopfer K, Schneiderbanger K, Schmidt M and Löhrs U (1996) Apoptosis and immunohistochemical bcl-2 expression in colorectal adenomas and carcinomas. Aspects of carcinogenesis and prognostic significance. *Cancer* **77**: 255–264

Bargou RC, Daniel PT, Mapara MY, Bommert K, Wagener C, Kallinich B, Royer HD and Dorken B (1995) Expression of the bcl-2 gene family in normal and malignant breast tissue: low bax-alpha expression in tumor cells correlates with resistance towards apoptosis. *Int J Cancer* **60**: 854–859

Bargou RC, Wagener C, Bommert K, Mapara MY, Daniel PT, Arnold W, Dietel M, Guski H, Feller A, Royer HD and Dorken B (1996) Overexpression of the death-promoting gene bax-alpha which is downregulated in breast cancer restores sensitivity to different apoptotic stimuli and reduces tumor growth in SCID mice. *J Clin Investigation* **97**: 2651–2659

Berney CR, Downing SR, Yang J-L, Russell PJ and Crowe PJ (2000) Evidence for post-transcriptional down-regulation of the apoptosis-related gene bcl-2 in human colorectal cancer. *J Pathol* **191**: 15–20

Bonetti A, Zaninelli M, Leone R, Cetto GL, Pelosi G, Biolo S, Menghi A, Manfrin E, Bonetti F and Piubello Q (1998) bcl-2 but not p53 expression is associated

 © 2001 Cancer Research Campaign with resistance to chemotherapy in advanced breast cancer. *Clin Cancer Res* **4**: 2331–2336

Bubb VJ, Curtis LJ, Cunningham C, Dunlop MG, Carothers AD, Morris RG, White S, Bird CC and Wyllie AH (1996) Microsatellite instability and the role of hMSH2 in sporadic colorectal cancer. *Oncogene* **12**: 2641–2649

Campos L, Rouault JP, Sabido O, Oriol P, Roubi N, Vasselon C, Archimbaud E, Magaud JP and Guyotat D (1993) High expression of bcl-2 protein in acute myeloid leukemia cells is associated with poor response to chemotherapy. *Blood* **81**: 3091–3096

Clark JW (1997) Perspectives on new chemotherapeutic agents in the treatment of colorectal cancer. *Semin Oncol* **24** (suppl 18): s18–24

Costa A, Marasca R, Valentinis B, Savarino M, Faranda A, Torelli G and Silvestrini R (1995) p53 gene point mutations in relation to p53 nuclear protein accumulation in colorectal cancers. *J Pathol* **176**: 45–53

Costa A, Doci R, Mochen C, Bignami P, Faranda A, Gennari L and Silvestrini R (1997) Cell proliferation-related markers in colorectal liver metastases: correlation with patient prognosis. *J Clin Oncol* **15**: 2008–2014

Costa A, Licitra L, Veneroni S, Daidone MG, Grandi C, Cavina R, Molinari R and Silvestrini R (1998) Biological markers as indicators of pathological response to primary chemotherapy in oral cavity cancers. *Int J Cancer* (Pred Oncol) **79**: 619–623

Daidone MG, Veneroni S, Benini E, Tomasic G, Coradini D, Mastore M, Brambilla C, Ferrari L and Silvestrini R (1999) Biological markers as indicators of response to primary and adjuvant chemotherapy in breast cancer. *Int J Cancer* (Pred Oncol) **84**: 580–586

De Angelis PM, Stokke T, Thorstensen L, Lothe RA and Clausen OPF (1998) Apoptosis and expression of bax, bcl-x, and bcl-2 apoptotic regulatory proteins in colorectal carcinomas, and associations with p53 genotype/phenotype. *J Clin Pathol: Mol Pathol* **51**: 254–261

Dive C and Hickman JA (1991) Drug-target interactions: only the first step in the commitment to a programmed cell death? *Br J Cancer* **64**: 192–196

Eastman A (1990) Activation of programmed cell death by anticancer agents: cisplatin as a model system. *Cancer Cells* **2**: 275–280

Farczadi E, Szanto J, Kaszas I, Benyo I, Bodnar Z, Szlobodnyik J and Szende B (1999) Changes in apoptotic and mitotic activity in rectal carcinoma after short-term cytostatic therapy as possible predictive factors. *Neoplasma* **46**: 219–223

Forster S, Sattler HP, Hack M, Romanakis K, Rohde V, Seitz G and Wullich B (1998) Microsatellite instability in sporadic carcinomas of the proximal colon: association with diploid DNA content, negative protein expression of p53, and distinct histomorphologic features. *Surgery* **123**: 13–18

Fracchiolla NS, Capaccio P, Carboni N, Pagliari AV, Neri A, Ronchett D, Pruner G, Silvotti MG, Pignataro L, Buffa R and Broich G (1999) Immunohistochemical and molecular analysis of bax, bcl-2 and p53 genes in laryngeal squamous cell carcinomas. *Anticancer Res* **19**: 1043–1052

Gasparini G, Bevilacqua P, Bonoldi E, Testolini A, Galassi A, Verderio P, Boracchi P, Guglielmi RB and Pezzella F (1995) Predictive and prognostic markers in a series of patients with head and neck squamous cell invasive carcinoma treated with concurrent chemoradiation therapy. *Clin Cancer Res* **1**: 1375–1383

Gavrieli Y, Sherman Y and Ben-Sasson SA (1992) Identification of programmed cell death in situ via specific labeling of nuclear DNA fragmentation. *J Cell Biol* **119**: 493–501

Hall PA (1999) Assessing apoptosis: a critical survey. *Endocrine-Related Cancer* **6**: 3–8

Homma A, Furuta Y, Oridate N, Nakano Y, Dohashi G, Yagi K, Nagahashi T, Fukuda S, Inoue K and Inuyama Y (1999) Prognostic significance of clinical parameters and biological markers in patients with squamous cell carcinoma of the head and neck treated with concurrent chemoradiotherapy. *Clin Cancer Res* **5**: 801–806

Johnston PG, Liang CM, Henry S, Chabner BA and Allegra CJ (1991) Production and characterization of monoclonal antibodies that localize human thymidylate synthase in the cytoplasm of human cells and tissues. *Cancer Res* **51**: 6668–6676

Kawasaki H, Altieri DC, Lu C-D, Toyoda M, Tenjo T and Tanigawa N (1998) Inhibition of apoptosis by survivin predicts shorter survival rates in colorectal cancer. *Cancer Res* **58**: 5071–5074

Krajewski S, Blomqvist C, Franssila K, Krajewska M, Wasenius V-M, Niskanen E, Nordling S and Reed JC (1995) Reduced expression of pro-apoptotic gene bax is associated with poor response rates to combination chemotherapy and shorter survival in women with metastatic breast adenocarcinoma. *Cancer Res* **55**: 4471–4478

Langlois NE, Lamb J, Eremin O and Heys SD (1997) Apoptosis in colorectal carcinoma occurring in patients aged 45 years and under: relationship to prognosis, mitosis, and immunohistochemical demonstration of p53, c-myc and bcl-2 products. *J Pathol* **182**: 392–397

Liang JT, Chang KJ, Chen JC, Lee CC, Cheng YM, Hsu HC, Chien CT and Wang SM (1999) Clinicopathologic and carcinogenetic appraisal of DNA replication error in sporadic T3N0M0 stage colorectal cancer after curative resection. *Hepato-Gastroenterol* **46**: 883–890

Lukish JR, Muro K, DeNobile J, Katz R, Williams J, Cruess DF, Drucker W, Kirsch I and Hamilton SR (1998) Prognostic significance of DNA replication errors in young patients with colorectal cancer. *Ann Surg* **227**: 51–56

Machiavelli M, Leone BA, Romero A, Rabinovich MG, Vallejo CT, Bianco A, Perez JE, Rodriquez R, Cuevas MA, Alvarez LA, Chacon R and Hannois A (1991) Advanced colorectal carcinoma. *Am J Clin Oncol* **14**: 211–217

Matsuura T, Fukuda Y, Fujitaka T, Nishisaka T, Sakatani T and Ito H (2000) Preoperative treatment with tegafur suppositories enhances apoptosis and reduces the intratumoral microvessel density of human colorectal carcinoma. *Cancer* **88**: 1007–1015

McDonnell TJ, Troncoso P, Brisbay SM, Logothetis C, Chung LW, Hsieh JT, Tu SM and Campbell ML (1992) Expression of the protooncogene bcl-2 in the prostate and its association with emergence of androgen-independent prostate cancer. *Cancer Res* **52**: 6940–6944

Nelson NJ (1998) Quest for new and better colon cancer treatments picks up steam. *J Natl Cancer Inst* **90**: 1858–1859

Newland RC, Dent OF, Lyttle MNB, Chapius PH and Bokey EL (1994) Pathologic determinants of survival associated with colorectal cancer with lymph node metastases. *Cancer* **73**: 2076–2082

Öfner D, Riehemann K, Maier H, Riedmann B, Nehoda H, Tötsch M, Böcker W, Jasani B and Schmid KW (1995) Immunohistochemically detectable bcl-2 expression in colorectal carcinoma: correlation with tumour stage and patient survival. *Br J Cancer* **72**: 981–985

Ogura E, Senzaki H, Yamamoto D, Yoshida R, Takada H, Hioki K and Tsubura A (1999) Prognostic significance of bcl-2, bcl-$x_{L/S}$, bax and bak expressions in colorectal carcinomas. *Oncol Reports* **6**: 365–369

Orlandi L, Bearzatto A, Abolafio G, De Marco C, Daidone MG and Zaffaroni N (1999) Involvement of bcl-2 and p21 waf1 proteins in response of human breast cancer cell clones to Tomudex. *Br J Cancer* **81**: 252–260

Paradiso A, Rabinovich M, Vallejo C, Machaivelli M, Romero A, Perez J, Lacava J, Cuevas MA, Rodriquez R, Leone B, Sapia MG, Simone G and De Lena M (1996) p53 and PCNA expression in advanced colorectal cancer: response to chemotherapy and long-term prognosis. *Int J Cancer* (Pred Oncol) **69**: 437–441

Paradiso A, Simone G, Petroni S, Leone B, Vallejo C, Lacava J, Romero A, Machiavelli M, De Lena M, Allegra CJ and Johnston PG (2000) Thymidylate synthase and p53 primary tumour expression as predictive factors for advanced colorectal cancer. *Br J Cancer* **82**: 560–567

Petrelli NJ (1995) It is time to stratify with prognostic factors for hepatic metastases. *J Clin Oncol* **13**: 2471–2472

Rampino N, Yamamoto H, Ionov Y, Li Y, Sawai H, Reed JC and Perucho M (1997) Somatic frameshift mutations in the BAX gene in colon cancers of the microsatellite mutator phenotype. *Science* **275**: 967–969

Schmitt CA and Lowe S (1999) Apoptosis and therapy. *J Pathol* **187**: 136–149

Silvestrini R, Veneroni S, Daidone MG, Benini E, Boracchi P, Mezzetti M, Di Fronzo G and Veronesi U (1994) bcl-2, a prognostic indicator strongly related to p53 in node-negative breast cancer. *J Natl Cancer Inst* **86**: 499–504

Silvestrini R, Daidone MG, Veneroni S, Benini E, Scarfone G, Zanaboni F, Villa A, Presti M, Danese S and Bolis G (1998) The clinical predictivity of biomarkers of stage III–IV epithelial ovarian cancers in a prospective randomized treatment protocol. *Cancer* **82**: 159–167

Sionov RV and Haupt Y (1999) The cellular response to p53: the decision between life and death. *Oncogene* **18**: 6145–6157

Sturm E, Köhne C-H, Wolff G, Petrowsky H, Hillebrand T, Hauptmann S, Lorenz M, Dörken B and Daniel PT (1999) Analysis of the p53/bax pathway in colorectal cancer: low bax is a negative prognostic factor in patients with resected liver metastases. *J Clin Oncol* **17**: 1364–1374

Sugamura K, Makino M and Kaibara N (1998) Apoptosis as a prognostic factor in colorectal carcinoma. *Surgery Today* **28**: 145–150

Sugura K, Makino M, Shirai H, Kimura O, Maeta M, Itoh H and Kaibara N (1997) Enhanced induction of apoptosis of human gastric carcinoma cells after preoperative treatment with 5-fluorouracil. *Cancer* **79**: 12–17

Tai Y-T, Lee S, Niloff E, Weisman C, Strobel T and Cannistra SA (1998) Bax protein expression and clinical outcome in epithelial ovarian cancer. *J Clin Oncol* **16**: 2583–2590

Takano Y, Saegusa M, Ikenaga M, Mitomi H and Okayasu I (1996) Apoptosis of colon cancer: comparison with Ki-67 proliferative activity and expression of p53. *J Cancer Res Clin Oncol* **122**: 166–170

Tang R, Wang YJ, Chen JS, Chang-Chien CR, Tang S, Lin SE, You YT, Hsu KC, Ho YS and Fan HA (1995) Survival impact of lymph node metastasis in TNM stage II carcinoma of the colon and rectum. *J Am Coll Surg* **180**: 705–712

© 2001 Cancer Research Campaign 

Tannapfel A, Nusslein S, Fietkau R, Katalinic A, Kockerling F and Wittekind C (1998) Apoptosis, cell proliferation, bax, bcl-2 and p53 status prior to and after preoperative radiochemotherapy for locally advanced rectal cancer. *Int J Radiat Oncol Biol Phys* **41**: 585–591

Teixeira C, Reed JC and Pratt MA (1995) Estrogen promotes chemotherapeutic drug resistance by a mechanism involving bcl-2 proto-oncogene expression in human breast cancer cells. *Cancer Res* **55**: 3902–3907

Tenjo T, Toyoda M, Okuda J, Wtanabe I, Yamamoto T, Tanaka K, Ohtani M, Nohara T, Kawasaki H and Tanigawa N (2000) Prognostic significance of $p27^{kip1}$ protein expression and spontaneous apoptosis in patients with colorectal adenocarcinomas. *Oncology* **58**: 45–51

Yamane N, Makino M and Kaibara N (1999) S-phase accumulation precedes apoptosis induced by preoperative treatment with 5-fluorouracil in human colorectal carcinoma cells. *Cancer* **85**: 309–317

Yunis JJ, Mayer MG, Arnesen MA, Aeppli DP, Oken MM and Frizzera G (1989) bcl-2 and other genomic alterations in the prognosis of large-cell lymphoma. *New Engl J Med* **320**: 1047–1054

 *© 2001 Cancer Research Campaign*
